# Prevalence and Outcome of Serum Autoantibodies in Chronic Hepatitis C Patients Undergoing Direct-Acting Antiviral Treatment

**DOI:** 10.3389/fimmu.2022.882064

**Published:** 2022-04-11

**Authors:** Ciro Romano, Olga Tortorella, Liliana Dalla Mora, Dario Di Stasio, Ausilia Sellitto, Luigi Elio Adinolfi, Aldo Marrone

**Affiliations:** ^1^Division of Internal Medicine, Department of Advanced Medical and Surgical Sciences, “Luigi Vanvitelli” University of Campania, Naples, Italy; ^2^Department of Precision Medicine, “Luigi Vanvitelli” University of Campania, Naples, Italy; ^3^Multidisciplinary Department of Medical and Dental Specialties, University of Campania “Luigi Vanvitelli”, Naples, Italy

**Keywords:** hepatitis C virus (HCV), direct-acting antivirals (DAAs), anti-nuclear antibodies (ANA), anti-smooth muscle antibodies (ASMA), anti-mitochondrial antibodies (AMA), autoantibodies

## Abstract

**Background:**

Chronic immune stimulation by hepatitis C virus (HCV) may cause occurrence of several autoantibodies in infected patients, with or without features of clinically overt autoimmune diseases. The recent introduction of direct-acting antivirals (DAAs) has dramatically changed the natural history of chronic HCV infection. The aim of this study was to assess the effects of DAA therapy on serum autoantibodies in chronic hepatitis C (CHC) patients.

**Methods:**

The medical records of 113 CHC patients were reviewed to assess autoantibody behavior following DAA-directed HCV eradication. Statistical analysis was performed to assess correlations between DAA treatment and autoantibody titers, HCV genotypes, and viral loads.

**Results:**

Anti-nuclear (ANA), anti-smooth muscle cell (ASMA) and anti-mitochondrial (AMA) antibody testing was available in 77 patients; 31 out of 77 patients (40%) had one or more serum autoantibodies prior to treatment. Measurement of autoantibody titers before and after HCV eradication was performed in 20 of 31 patients. DAA treatment significantly affected ANA and ASMA titers, leading to disappearance or reduction of autoantibody titers; conversely, AMA were not influenced by DAA treatment. No correlations were observed between autoantibody specificity and both HCV genotypes and viral loads at baseline. Likewise, serum autoantibody titers were independent of HCV genotypes.

**Conclusions:**

DAA-directed HCV clearance may interrupt chronic immune stimulation by removing the drive for autoantibody induction. The isolated persistence of autoantibodies in the small fraction of patients who did not show clearance following DAA treatment may require long-term vigilance.

## Introduction

Chronic hepatitis C represents an important cause of morbidity and mortality because of its widespread prevalence (1). Liver involvement is only one side of the coin, as extrahepatic manifestations may complicate the course of disease in over 70% of hepatitis C virus (HCV)-infected individuals (2–4). A wide variety of extrahepatic manifestations have been described in association with HCV infection, including lymphoproliferative disorders, cardiovascular disease, metabolic derangements, renal involvement, dermatologic manifestations, neuropsychiatric alterations, and autoimmune diseases (2–12). Many of the extrahepatic manifestations of HCV infection may be considered as the consequence of an aberrant immune response, among which mixed cryoglobulinemia is the most frequent and best studied (13). Indeed, since the immune response against HCV is not effective owing to the virus’ ability to modify its antigenicity, chronic engagement of initially activated and then exhausted immune cells may turn deleterious to the infected individual (14). Serological evidence of chronic immune stimulation is usually reflected by the occurrence of a wide variety of autoantibodies, including anti-nuclear antibodies (ANA), anti-smooth muscle antibodies (ASMA), rheumatoid factor, etc., which may be just the laboratory “signature” of this immune effort to clear the viral infection or even be accompanied by clinical features of autoimmunity (15). Recently, the introduction of direct-acting antivirals (DAAs) into the therapeutic armamentarium has revolutionized the treatment of HCV infection (16). Indeed, DAAs allow nearly all HCV-infected patients to obtain quick and curative resolution of the disease, with excellent treatment tolerability (16). Aims of this study were, therefore, to assess the prevalence of and DAAs impact on serum autoantibodies in a cohort of chronic hepatitis C patients referred to our dedicated outpatient clinic.

## Patients and Methods

### Study Participants and Data Collection

**Figure 1:**
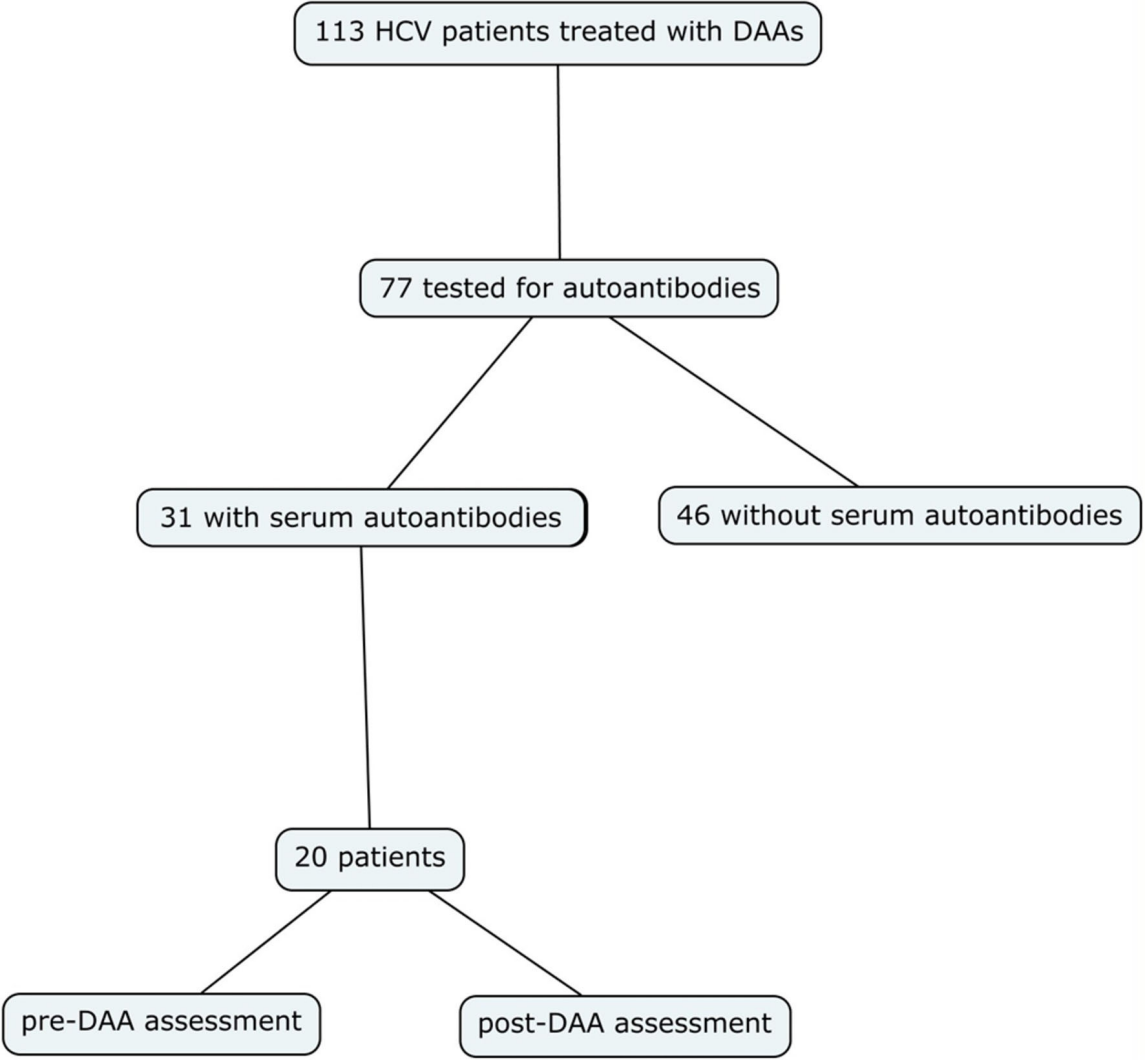
Patient recruitment diagram.

One hundred thirteen consecutive patients with chronic hepatitis C referred to our hepatology service for therapeutic assessment and eligible for treatment with DAAs were retrospectively evaluated. Hepatic fibrosis was noninvasively investigated by means of Fibroscan, according to Metavir classification (17, 18). Therapy for concurrent comorbidities was checked for pharmacologic interactions with DAAs (HEP Interactions, University of Liverpool) (19) and adjusted accordingly in case of known interference. Patients were treated with DAAs ± ribavirin (RBV) for 8, 12, or 24 weeks, according to established guidelines (20, 21) and based on local drug availability. All relevant data (clinical, biochemical, immunologic, and virologic) were collected at baseline (i.e., before starting DAA treatment), 2 weeks after starting treatment (T2) and every 4 weeks thereafter until treatment completion. Follow-up was scheduled at 12, 24, and 48 weeks following end of DAA treatment. HCV-RNA was measured by means of reverse transcriptase (RT)- polymerase chain reaction (PCR), according to standard protocols (sensitivity: <12 IU/ml), 12 weeks after completion of therapy. Autoantibodies were detected by means of indirect immunofluorescence on tissue slides; pattern and titer were used to specify positivity, according to the International Consensus on ANA Patterns (ICAP) criteria (22). Autoantibody titers before start of DAA treatment and 24 months after end of therapy were available in 64.5% of patients initially found to have serological autoimmunity (**Figure 1**). The study was carried out in accordance with the Declaration of Helsinki and its later amendments; because of its retrospective nature, formal consent was not required.

### Statistical Analysis

For statistical analysis, the titers of ANA, anti-mitochondrial antibodies (AMA), and ASMA were categorized as follows: a) negative; b) very low titer (positive at 1:40 dilution); c) low titer (positive at 1:80-1:160 dilution); d) moderate/high titer (positive at ≥1/320 dilution). Sign test was used to assess the median differences between baseline and the sustained virological response at 24 weeks (SVR24) titers of ANA, AMA, ASMA. Correlation significance between HCV genotype, viral load (log IU/ml) and presence of ANA, AMA and ASMA at baseline was calculated with the Spearman equation. Kruskal Wallis test was used to assess the relationship between ANA, AMA and ASMA titers and the different genotypes. Statistical analysis was performed using the SPSS21 statistical package. Level of significance was specified at *p <*0.05.

## Results

### Patient Characteristics

Baseline characteristics of patients are summarized in **Table 1**. Briefly, about two thirds of patients were aged ≥ 65 years, there was gender equivalence (∽1:1), and the chronic hepatitis/cirrhosis ratio was about 3:1. Genotype 1b was detected in more than half of patients (52%). Slightly more than one quarter of patients had been previously treated with interferon (IFN)-α.

**Table 1:** Baseline characteristics of HCV-infected patients undergoing DAA treatment.

Patients, no.	113
M/F	57 (50.4%)/56 (49.6%)
Age, mean (years)	66
Chronic hepatitis C, no.	87 (77%)
Cirrhosis, no.	26 (23%)
Previous IFN-α treatment, no.	30 (26.5%)
Pre-treatment HCV-RNA, median (range)	4.35 x 10^6^ UI/ml (1.3 x 10^2^ – 7.5 x 10^7^)
Pre-treatment Fibroscan, median (range)	10.9 kPa (3.7- 42.5)
HCV genotype, %	
1a	11
1b	52
2	29
3	6
4	2
Comorbidities (%)	
hypertension	39
cardiovascular	26
hematologic	20
gastrointestinal	20
endocrine	17
diabetes	15
dermatologic	13
rheumatologic	13
pulmonary	12
neurologic	12
psychiatric	10
cancer	5
sense organs	4

### Comorbidities

Due to a median age of 66 years, comorbidities were frequently detected in the study population. The most common condition associated with HCV infection was hypertension (39% of patients), followed by cardiovascular disease (26%), hematologic conditions (20%, with more than one third represented by non-Hodgkin lymphoma), gastrointestinal disease (20%). Diabetes was observed in 15% of patients. The complete list of comorbidities is shown in **Table 1**.

### Autoantibody Behavior Before and After Treatment With DAAs

**Figure 2:**
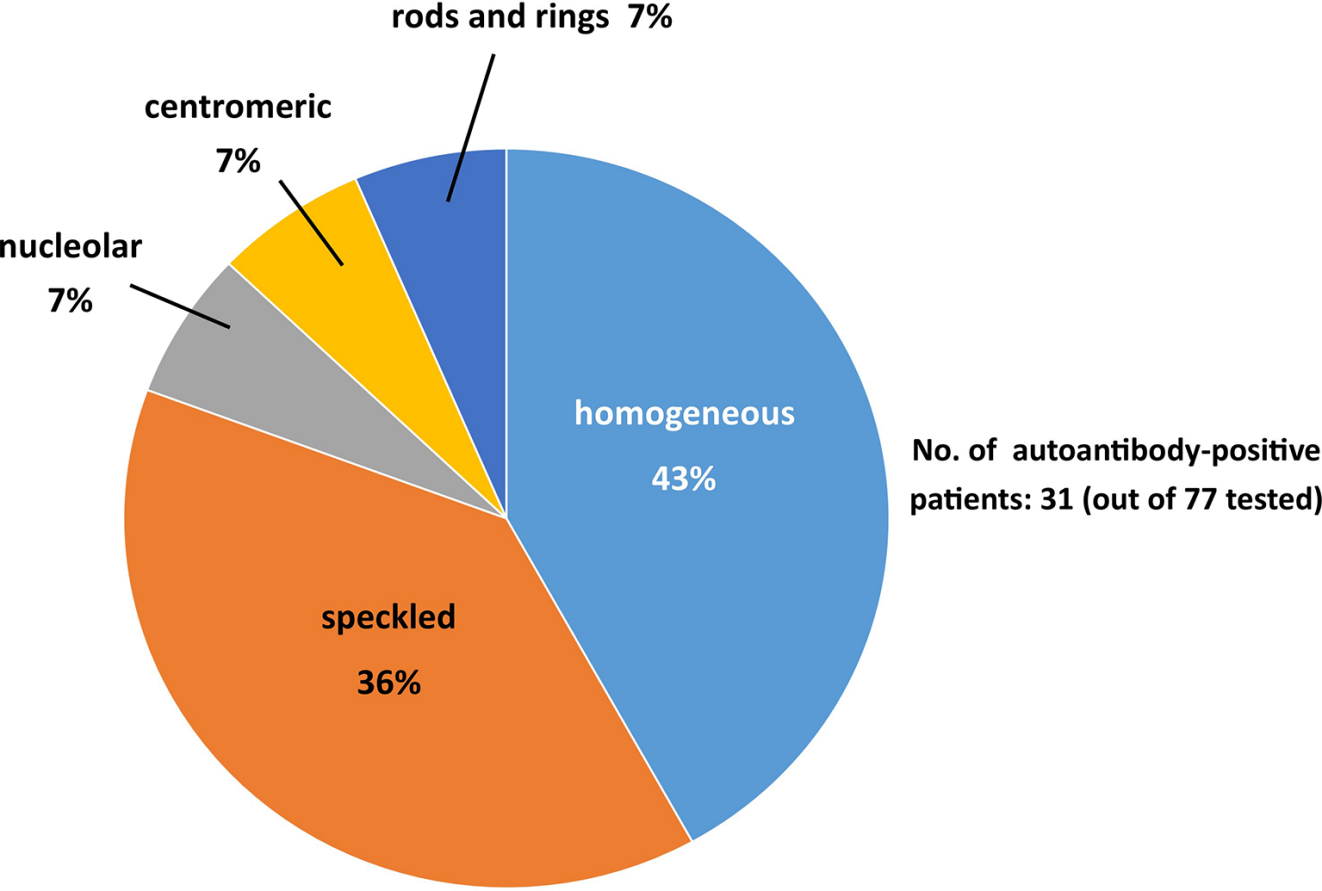
Prevalence of ANA patterns in HCV-infected patients.

Autoantibody testing was available in 77 out of 113 patients (**Figure 1**). Serum autoantibodies prevalence in our cohort of HCV-infected patients was 40.2% (31 out of 77 patients). Specifically, ANA, ASMA and AMA were detectable, alone or in combination, in 94%, 39%, and 10% of patients, respectively. **Figure 2** summarizes the prevalence of ANA patterns on immunofluorescence staining. ENA profile was negative in all patients, except in those with a centromeric pattern. Autoantibody testing was available before and after DAA treatment in 20 out of 31 patients who were initially found to have one or more serum autoantibodies. Clinical characteristics of the 20 patients are summarized in **Table 2**.

**Table 2:** Characteristics of the 20 patients with pre- and post-DAA treatment autoantibody testing.

M/F	5/15	
Age		
*mean±standard deviation, range (years)*	71.65±11.1, 40-87	
HCV genotypes	1a	3 patients
	1b	9 patients
	2	8 patients
Chronic hepatitis/cirrhosis *(no.)*	13/7	
Fibroscan, pre-/post-DAA treatment		
*mean±standard deviation (kPa)*	12.82±7.6/9.7±4.8 (*p*=ns)	
Previous IFN-α + ribavirin treatment *(no.)*	8	
Overt clinical autoimmunity *(no.)*	3 patients	
	(2 with primary biliary cirrhosis, 1 with autoimmune thyroiditis)	

**Figure 3:**
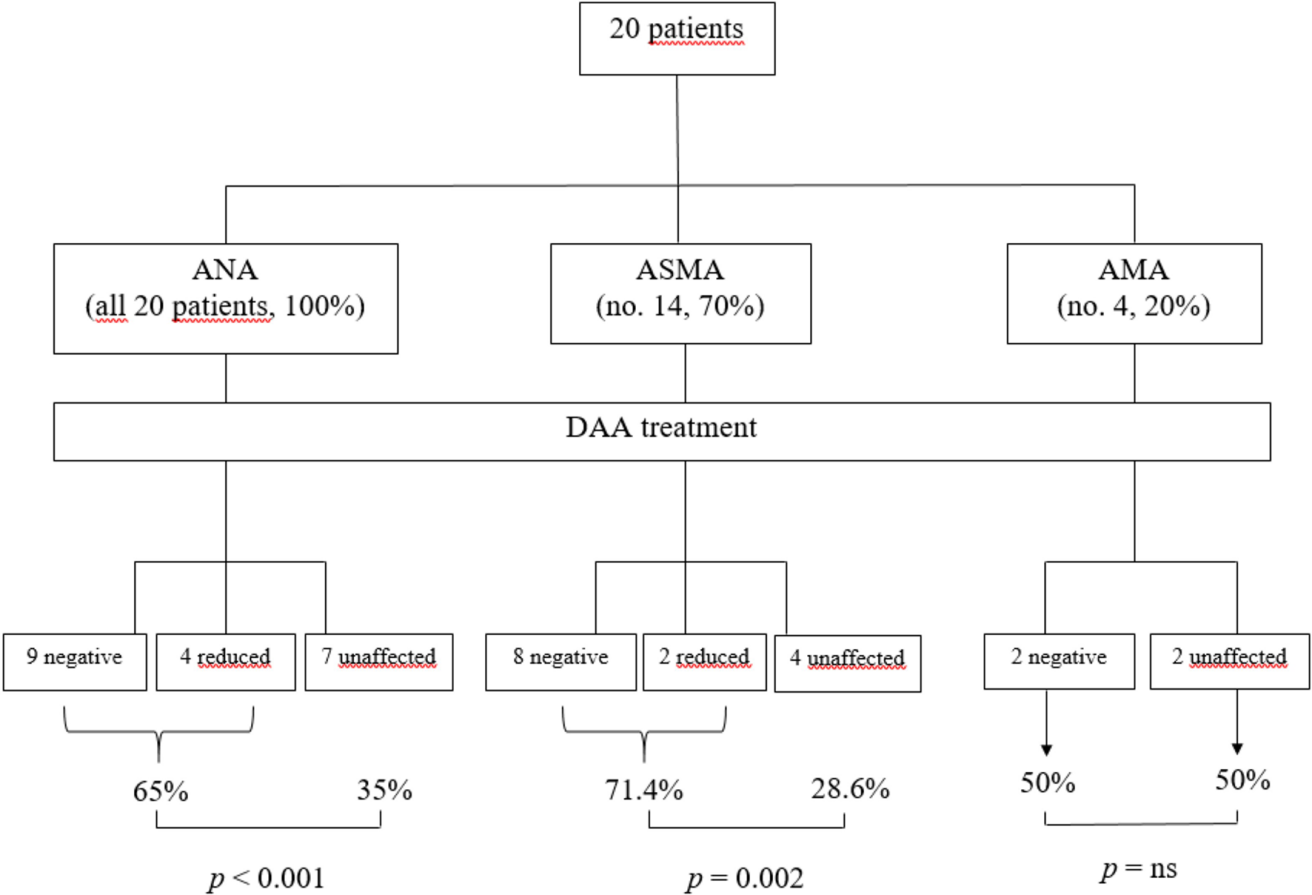
Outcome of serological autoimmunity after DAA treatment.

The behavior of serum autoantibodies was significantly affected by DAA-mediated HCV eradication. Briefly, DAA treatment significantly impacted on ANA positivity in 13 out of 20 patients (65% of tested subjects, *p*<0.001), with 9 patients turning negative, 1 patient displaying a reduction to a very low titer (from 1:80 to 1:40), and 3 patients (2 with 1:640 and 1 with 1:320, respectively) showing a reduction to a low titer (1:80). Seven patients did not show any change in ANA titers (**Figure 3**).

Likewise, ASMA titers were significantly modified by DAA treatment (*p*=0.002) in 71.4% of patients: out of 14 positive serum samples, eight turned negative, while 2 patients had their ASMA titers decreased from 1:320 to 1:160 and to 1:80, respectively (**Figure 3**).

Conversely, AMA titers were not significantly affected by DAA treatment (*p*=0.500): only two patients tested negative at the end of anti-viral treatment. However, it should be noted that only 4 patients showed autoantibody positivity at baseline (**Figure 3**).

There was no correlation between autoantibody specificity and both HCV genotypes (ANA: rs=0.231, *p*=0.327; AMA: rs=-0.182, *p*=0.443; ASMA: rs=-0.134, *p*=0.897) and viral loads (ANA: rs=0.351, *p*=0.154; AMA: rs=-0.187, *p*=0.458; ASMA: rs=-0.012, *p*=0.572) at baseline. Likewise, no differences were recorded between serum autoantibody titers (ANA: *p*=0.275; AMA: *p*=0.737; ASMA *p*=0.175) and the different HCV genotypes.

### Type of Treatment

Treatment regimens are summarized in **Table 3**. Sofosbuvir/velpatasvir was used more frequently. RBV was associated to DAAs in 11% of cases.

**Table 3:** DAA regimens used in the patient population.

Sofosbuvir/Velpatasvir	33/93 (36%)
Paritaprevir/Ritonavir/Ombitasvir +Dasabuvir	13/93 (14%)
Glecaprevir/Pibrentasvir	13/93 (14%)
Elbasvir/Grazoprevir	14/93 (15%)
Sofosbuvir/Ledipasvir	9/93 (10%)
Sofosbuvir + Daclatasvir	3/93 (3%)
Sofosbuvir + Simeprevir	2/93 (2%)
Sofosbuvir/Velpatasvir/Voxilaprevir	3/93 (3%)
Sofosbuvir	3/93 (3%)

### Follow-Up

Relapse of HCV infection was observed in only 4% of patients, who required a further course with DAAs. The remaining 96% of patients achieved a sustained virological response at 12 months. Patients with persistence of autoantibodies (ANA and/or ASMA and/or AMA) did not show clinically overt autoimmune diseases at the latest follow-up (at least 35 months after sustained virological response), excluding two patients with previously known primary biliary cholangitis overlapping HCV infection and a female patient with anti-centromere autoantibodies suspected of having a concurrent early limited variant of systemic sclerosis.

## Discussion

In this retrospective study, we aimed at determining the impact of DAA treatment on serum autoantibodies in chronic hepatitis C patients. Specifically, we showed that a significant proportion of patients with serum autoantibodies turned out to be negative or displayed a significant titer reduction following successful eradication of HCV infection. Indeed, nearly two thirds of ANA-positive patients and more than 70% of ASMA-positive patients did not either show detectable autoantibodies or had serum titers reduced at the 24-week follow-up after DAA treatment initiation. These observations are in line with the notion that chronic immune stimulation induced by HCV may lead to dysregulated immune responses possibly resulting in frank autoimmune phenomena (23–25). Consistently, HCV clearance may stop chronic immune stimulation thus reverting the likelihood of subclinical or overt autoimmunity (26). The significance of persistently positive serum autoantibodies in the small fraction of patients who did not show disappearance of these autoreactive immunoglobulins following DAA treatment may require careful long-term follow-up (27, 28); in these patients, autoreactivity may not be linked to HCV infection (i.e., is independent of) or it may have become disengaged from the triggering viral infection. This latter hypothesis is supported by the observation of persistent cryoglobulinemia in a significant proportion of patients with HCV-related cryoglobulinemic vasculitis, despite achievement of a sustained viral response following DAA treatment (29, 30). Moreover, relapses of cryoglobulinemic vasculitis in these patients have been reported within 2 years of a sustained viral response to DAA therapy (30). Thus, a vigilant eye must be kept in order to recognize early in their development possible extrahepatic autoimmune manifestations in patients who still display positive autoantibody titers despite successful DAA therapy (27, 28). Notwithstanding, at the latest follow-up (at least 35 months after sustained viral clearance), we have not recorded yet any frank autoimmune diseases in our cohort of HCV-eradicated patients with persistence of serum autoantibodies. Unfortunately, at the moment, it is not clear why certain patients remain positive for serum autoantibodies long after SVR as opposed to others who become quickly seronegative after sustained HCV clearance. As there is no formal need for therapy in the former group in the absence of clinical manifestations, long-term follow-up should be the recommended strategy.

There are only a few reports in the literature addressing the role of DAAs in clearing autoimmune serology in chronic hepatitis C patients and our results are in full accordance with these previous investigations (27, 28, 31). As only small case series have been published on this topic thus far (27, 28, 31), our report further contributes to and strengthens the notion of HCV being responsible for undermining immune tolerance in chronically infected patients. Besides, more than half of the patients (no. 11) have been followed up for more than 5 years, while for the remaining ones at least nearly three years (35 months) have thus far elapsed since end of DAA treatment. There are no other papers in the literature reporting such a long follow-up.

It should be noted that the rate of DAA-induced autoantibody clearance could have been even higher in our experience, had we excluded from the analyses patients with centromeric and rod and ring patterns. The centromeric pattern is highly suggestive of systemic sclerosis, limited variant (32), whereas the rod and ring pattern is known to be associated with a previous treatment with IFN-α and RBV (33, 34). Thus, we would have not expected clearance of these antibodies following DAA treatment anyway, as they were not closely related to HCV infection.

Clearly, this study has some limitations. First, because of the relatively small patient population, our results need to be confirmed in larger cohorts; second, the study was conducted in a single center, therefore, validation in different geographic areas would be desirable; finally, due to the retrospective nature of the study, potential bias may have not been recognized.

In conclusion, DAA did favorably impact on serum autoantibodies in our patient population. As our HCV-cleared patients with persistently positive autoantibody serology have not been shown to have developed clinical autoimmunity despite the quite long follow-up thus far, we would be tempted to affirm the benign nature of these alterations (provided cryoglobulins are excluded from the evaluation)!; however, to definitively confirm the benign outcome of patients with persistent serum autoantibodies, we would cautiously suggest waiting for a further follow-up extension.

## Data Availability Statement

Raw data are available from the corresponding author upon reasonable request.

## Ethics Statement

The study was carried out in accordance with the Declaration of Helsinki and its later amendments; because of its retrospective nature, formal consent was not required.

## Author Contributions

CR and AM provided substantial contributions to the conception and design of the study and in the writing of the article; AM, CR, OT, AS, and LDM contributed to the acquisition and the interpretation of the data; LEA and DDS carried out the statistical analyses. All authors were involved in the critical revision of the manuscript and approved the final version of the article.

## Funding

Funds from open access publication fees come from the Department of Advanced Medical and Surgical Sciences.

## Conflict of Interest

The authors declare that the research was conducted in the absence of any commercial or financial relationships that could be construed as a potential conflict of interest.

## Publisher’s Note

All claims expressed in this article are solely those of the authors and do not necessarily represent those of their affiliated organizations, or those of the publisher, the editors and the reviewers. Any product that may be evaluated in this article, or claim that may be made by its manufacturer, is not guaranteed or endorsed by the publisher.
